# Socio-Demographic and Prenatal Care Factors Associated with TORCH Screening During Pregnancy in Romania: A Cross-Sectional Study

**DOI:** 10.3390/healthcare14142087

**Published:** 2026-07-13

**Authors:** Mihaela Corina Radu, Laura Ioana Chivu, Letitia Draghici Goraneanu, Justin Aurelian, Raluca Elena Hanu, Loredana Sabina Cornelia Manolescu

**Affiliations:** 1 Department of Nursing, Faculty of Midwifery and Nursing, “Carol Davila” University of Medicine and Pharmacy, 020021 Bucharest, Romania; 2 Emergency Hospital County, Str. Mihai Bravu No. 106, 100409 Ploieşti, Romania; goraneanu.letitia@yahoo.com; 3 Department of Pathophysiology, Faculty of Midwifery and Nursing, “Carol Davila” University of Medicine and Pharmacy, 050474 Bucharest, Romania; 4 ”Prof Dr. Th. Burghele” Clinical Hospital, 061344 Bucharest, Romania; 5 CMI Dr. Nicolau, 19 Dinicu Golescu Boulevard, 010865 Bucharest, Romania; raluca_hanu@yahoo.com; 6 Department of Microbiology, Parasitology and Virology, Faculty of Midwives and Nursing, “Carol Davila” University of Medicine and Pharmacy, 020021 Bucharest, Romania; loredana.manolescu@umfcd.ro

**Keywords:** TORCH infections, prenatal screening, pregnancy, congenital infections, maternal health, prenatal care, health literacy, Romania, public health, serological testing

## Abstract

**Highlights:**

**What are the main findings?**

- Approximately 75.6% of pregnant women reported undergoing serological testing for at least one infection included in the TORCH complex, while only about half reported a complete TORCH panel.
- Lower educational level, unemployment, and multiparity were associated with lower uptake of TORCH screening, whereas participation in prenatal education programs was associated with increased testing utilization.

**What are the implications of the main findings?**

- Socio-educational inequalities may significantly influence access to preventive prenatal investigations, even in settings with available prenatal medical services.
- Strengthening prenatal education programs and improving equitable access to standardized congenital infection screening may contribute to better maternal–fetal outcomes and a reduction in preventable perinatal complications.

**Abstract:**

**Background:** Congenital infections included in the TORCH complex remain an important cause of fetal and neonatal morbidity and mortality, being associated with miscarriage, intrauterine growth restriction, congenital malformations, neurological impairment, and long-term developmental sequelae. Prenatal serological screening may contribute to the early identification of maternal infections and facilitate preventive and therapeutic interventions. However, data regarding the utilization of TORCH screening and associated socio-demographic determinants in Romania remain limited. **Objective:** This study aimed to evaluate the self-reported uptake of prenatal serological testing for one or more infections included in the TORCH complex, particularly *Toxoplasma gondii*, rubella virus, cytomegalovirus (CMV), herpes simplex virus (HSV), and syphilis, and to identify socio-demographic, obstetrical, and prenatal care-related factors associated with TORCH testing among pregnant women in Romania. **Materials and Methods:** A cross-sectional observational study was conducted using an online self-administered questionnaire completed by 1301 pregnant women from Romania. Data collection was performed between August 2022 and March 2023 through digital platforms, including social media and pregnancy-related forums. The primary outcome was self-reported performance of serological testing for at least one TORCH-related infection during pregnancy. Associations between explanatory variables and TORCH testing were evaluated using chi-square tests and multivariable binary logistic regression models. Odds ratios (ORs) and 95% confidence intervals (95% CIs) were calculated. **Results:** Overall, 75.6% of participants reported undergoing serological testing for at least one infection included in the TORCH complex during pregnancy, while 49.3% reported a complete TORCH panel. The most frequently reported investigations were for *Toxoplasma gondii* (92.8%) and rubella virus (89.9%), whereas HSV testing was less commonly reported (42.5%). Lower educational level was the strongest independent factor associated with reduced likelihood of TORCH testing (adjusted OR = 0.08; 95% CI: 0.03–0.19; *p* < 0.001) (adjusted OR—aOR). Unemployment status (aOR = 0.70; 95% CI: 0.50–0.99; *p* = 0.045) and multiparity (aOR = 0.62; 95% CI: 0.49–0.77; *p* < 0.001) were also associated with lower testing uptake. In contrast, participation in prenatal education programs was associated with increased likelihood of TORCH testing (aOR = 1.37; 95% CI: 1.04–1.80; *p* = 0.024). The number of prenatal consultations was not independently associated with testing uptake. **Conclusions:** The uptake of prenatal serological screening for congenital infections (assessed using an expanded Romanian panel that includes hepatitis B and HIV in addition to the classical TORCH agents) in Romania appears to be influenced predominantly by socio-educational and behavioral factors rather than by the quantitative utilization of prenatal care services alone. Given the online recruitment strategy and the predominantly urban and highly educated sample, the reported uptake rates may overestimate population-level coverage. Significant inequalities in access to preventive prenatal investigations were observed, particularly among women with lower educational and socio-economic status. Strengthening prenatal education programs and improving equitable access to standardized prenatal screening may contribute to optimizing congenital infection prevention and maternal–fetal health outcomes.

## 1. Introduction

Congenital infections included in the TORCH complex—*Toxoplasma gondii*, rubella virus, cytomegalovirus (CMV), herpes simplex virus (HSV), syphilis, and other infections relevant during the perinatal period—represent a major cause of fetal and neonatal morbidity and mortality worldwide [1,2,3]. These infections are associated with miscarriage, intrauterine fetal death, intrauterine growth restriction, prematurity, severe congenital malformations, and long-term neurological complications [1,2,3,4]. The clinical impact of congenital infections extends beyond the neonatal period and is associated with neurological disabilities, hearing and visual impairment, neurodevelopmental disorders, and increased healthcare and long-term social support costs [2,3,4,5].

In recent years, interest in the prevention of congenital infections has increased considerably, particularly in the context of persistent regional differences in the prevalence of TORCH infections and access to prenatal screening [6,7]. In Eastern Europe, epidemiological data suggest a still relevant burden of congenital toxoplasmosis, congenital CMV infection, and congenital syphilis, alongside significant inequalities in access to prenatal care and preventive healthcare services [7,8,9]. Although rubella vaccination has significantly reduced the incidence of congenital rubella syndrome in many regions, preventable congenital infections continue to represent an important public health concern, particularly among socio-economically vulnerable populations [6,7,8,9].

Prenatal serological screening enables the identification of susceptible women or recent maternal infections and may facilitate appropriate fetal monitoring, prenatal counseling, and early therapeutic interventions when indicated [4,10]. However, TORCH screening strategies vary considerably across international guidelines and healthcare systems. Some recommendations support universal screening for certain infections, whereas others promote selective testing based on clinical risk factors, regional epidemiological context, and cost-effectiveness assessments [10,11,12]. This heterogeneity reflects differences in infection prevalence, organization of prenatal healthcare services, and the level of available evidence regarding the impact of routine screening on perinatal outcomes [11,12].

In addition, access to prenatal screening may be influenced by socio-demographic factors, educational level, utilization of prenatal healthcare services, health literacy, and characteristics of the healthcare system [13,14]. Educational interventions and programs aimed at improving prenatal awareness have demonstrated favorable effects on screening participation and the adoption of preventive behaviors during pregnancy [13,14,15,16,17]. Nevertheless, in Romania and other Eastern European countries, data regarding TORCH screening utilization and the factors associated with testing uptake remain limited, particularly at regional and community levels [7,14].

In Romania, prenatal care is regulated by the Ministry of Health through the National Program for Maternal and Child Health. Current national guidelines recommend serological screening for syphilis, hepatitis B, and HIV as mandatory components of prenatal surveillance for all pregnant women, while testing for Toxoplasma gondii, rubella, CMV, and HSV is recommended but not universally mandated and is largely dependent on clinical judgment and regional practice. There is no uniform national protocol requiring a complete TORCH panel for all pregnancies, resulting in significant variability in screening practices across healthcare settings and regions. In this context, the observed uptake rate of 75.6% for at least one TORCH-related investigation and 49.3% for a complete TORCH panel should be interpreted against a background of non-standardized recommendations rather than a universal screening mandate. These figures highlight a substantial gap between existing guidance and actual clinical practice [14,17,18].

Recent studies have further emphasized the heterogeneity of TORCH screening practices and their determinants. A 2022 practice guideline issued by the International Society of Ultrasound in Obstetrics and Gynecology (ISUOG) demonstrated that congenital CMV infection remains the leading infectious cause of non-genetic sensorineural hearing loss, underscoring the need for systematic prenatal serological surveillance [19]. A 2023 systematic review highlighted persistent under-screening for congenital toxoplasmosis and syphilis in low- and middle-income countries, attributing this gap to socio-economic barriers and insufficient prenatal care infrastructure [14]. In the Romanian context, significant disparities in prenatal screening coverage between urban and rural populations have been documented, with educational level and healthcare proximity identified as the principal determinants of access to preventive serological testing [14]. Moreover, a multinational European cohort study confirmed that maternal health literacy and participation in structured prenatal education programs independently predicted higher rates of TORCH panel completion, an observation directly relevant to the Romanian setting [20].

Despite this growing body of evidence, a critical research gap persists regarding the socio-demographic and behavioral determinants of TORCH screening uptake in Romania specifically. Existing national data are fragmented, rely predominantly on administrative records rather than patient-reported outcomes, and do not systematically examine the independent contributions of educational level, occupational status, parity, and participation in prenatal education to serological testing behavior. Furthermore, no previous Romanian study has applied multivariable logistic regression to disentangle the relative contributions of these factors while controlling for prenatal care utilization frequency. The present study was designed to address this gap by providing population-level evidence on self-reported TORCH screening coverage and its determinants in a large, geographically diverse sample of pregnant women in Romania. Available data from other Eastern European countries similarly document limited and heterogeneous TORCH screening coverage. Existing reports indicate that uptake rates for individual congenital infection investigations vary considerably across the region, with educational level and healthcare access consistently identified as key determinants, although direct comparisons are hampered by differences in national screening protocols and data collection methods [6,7]. In Romania specifically, the systematic review collated seroprevalence data on TORCH agents across Romanian regions, documenting significant geographical variability in infection rates, but did not examine individual-level socio-demographic predictors of serological testing uptake using patient-reported data [14]. The present study addresses this specific gap by providing the first large-scale, multivariable analysis of self-reported TORCH screening behavior among pregnant women in Romania, enabling direct identification of the population subgroups most at risk of insufficient prenatal infectious disease screening.

The aim of the present study was to evaluate the self-reported coverage of prenatal serological screening for selected infections included in the TORCH complex, particularly *Toxoplasma gondii*, rubella virus, CMV, HSV, and syphilis, in a regional cohort of pregnant women in Romania. In addition, the study aimed to analyze the associations between socio-demographic characteristics, obstetrical factors, prenatal care utilization, and the self-reported performance of TORCH testing. Using a cross-sectional design based on an online questionnaire and multivariable logistic regression analyses, the study sought to explore patterns of access to prenatal infectious disease screening and to identify population groups potentially vulnerable to reduced utilization of preventive maternal healthcare services.

## 2. Materials and Methods

### 2.1. Study Design

A cross-sectional observational study based on a self-administered online questionnaire was conducted among pregnant women in Romania. The study aimed to evaluate the self-reported uptake of serological screening for one or more infections included in the TORCH complex during pregnancy and to analyze the associations between socio-demographic characteristics, obstetrical factors, prenatal care utilization, and the performance of these preventive investigations. Data collection was performed between August 2022 and March 2023.

The study reporting followed the *Strengthening the Reporting of Observational Studies in Epidemiology* (STROBE) recommendations for observational studies. Given the cross-sectional design and the self-reported nature of the collected data, the findings allow the identification of statistical associations but do not permit the establishment of causal relationships between the investigated variables and TORCH testing uptake.

### 2.2. Participants and Recruitment

The study population included 1301 pregnant women who completed the online questionnaire during the data collection period. Participants were recruited through the distribution of an electronic link on frequently used digital platforms, including Facebook, Instagram, and forums dedicated to pregnancy and maternity in Romania.

Participation in the study was voluntary, anonymous, and confidential. Before accessing the questionnaire, participants received information regarding the aim of the research, the voluntary nature of participation, and personal data protection.

Data were collected exclusively online, an approach that facilitated participant access to the study and enabled the inclusion of pregnant women from multiple regions of Romania, thereby contributing to the geographical diversity of the sample. However, this recruitment method may be associated with a certain degree of selection bias, as participation depended on internet access and willingness to complete an online questionnaire. Consequently, women with higher levels of digital literacy and a greater interest in maternal health may be overrepresented in the study sample. This recruitment strategy may limit the generalizability of the findings to the overall population of pregnant women in Romania, particularly to those from the most socio-economically vulnerable strata or with limited digital access—groups that are precisely those most likely to have lower TORCH screening uptake. Consequently, the observed uptake rate of 75.6% should be interpreted with caution and may represent an overestimate of the true population-level coverage of TORCH screening among pregnant women in Romania.

Furthermore, the findings should be interpreted in light of the fact that information regarding TORCH testing was self-reported and was not validated against medical records or clinical registries. Nevertheless, self-administered questionnaires are widely used in population-based epidemiological research and provide valuable insights into health-related behaviors and experiences associated with prenatal care.

Despite these limitations, the large sample size and the inclusion of participants from different regions of the country provide valuable information regarding TORCH screening utilization among pregnant women in Romania and allow the identification of socio-demographic and behavioral factors associated with access to preventive maternal healthcare services.

Participants completed the questionnaire independently, without direct involvement of the researchers. All analyzed information was self-reported and could not be verified through medical records or clinical registries, which may imply the possibility of recall or reporting bias.

Inclusion criteria consisted of confirmed pregnancy, attendance of prenatal monitoring, and complete responses to questionnaire items relevant to TORCH testing. Incomplete, duplicate, or inconsistent responses that could affect the validity of the statistical analyses were excluded. The final analysis included only complete cases (*complete-case analysis*).

Specifically, the inclusion criteria were: confirmed pregnancy at the time of questionnaire completion; currently under prenatal monitoring by a healthcare provider; residing in Romania; aged 18 years or older; and provision of electronic informed consent prior to accessing the questionnaire. The exclusion criteria were: incomplete responses to one or more questionnaire items relevant to the primary outcome or to any explanatory variable included in the analysis; duplicate submissions identified through response pattern analysis; logically inconsistent responses (e.g., reported gestational age incompatible with stated obstetrical history); and withdrawal of consent before questionnaire completion. The final analysis was restricted to complete cases, with no imputation of missing values.

The minimum required sample size was estimated a priori using the binary proportion formula for cross-sectional studies, assuming a conservative TORCH screening prevalence of 50%, a precision margin of ±3%, and a significance level of α = 0.05 (z = 1.96), yielding a minimum of *n* = 1068 participants. Accounting for an anticipated non-response rate of 15–20%, a recruitment target of at least 1250 complete responses was established. The final analytical sample of 1301 participants exceeds this threshold and provides adequate statistical power for the multivariable analyses performed.

### 2.3. Variables and Questionnaire Administration

The questionnaire was administered electronically through a dedicated online platform (Google Forms). Access was provided via a link embedded in digital announcements distributed through social media platforms and pregnancy-related forums, targeting pregnant women across multiple regions of Romania. The instrument comprised eight thematic sections covering: socio-demographic characteristics; obstetrical history and current pregnancy details; prenatal care utilization; participation in prenatal education; self-reported serological testing for TORCH-related infections; knowledge and attitudes regarding congenital infections; sources of health information; and prior experience with TORCH-related complications. This structured administration ensures conceptual coherence between the variables collected and the outcomes evaluated in the present analysis.

The main objective communicated to participants was the evaluation of serological screening for congenital infections included in the TORCH complex and the identification of factors associated with the utilization of this type of prenatal screening.

The primary outcome variable was the self-reported performance of serological testing for at least one infection included in the TORCH complex during pregnancy. The questionnaire included items regarding testing for relevant congenital infections included in the TORCH panel. Participants were informed within the questionnaire that a complete TORCH panel referred to serological testing for the main congenital infections included in this complex, namely toxoplasmosis, rubella, CMV infection, HSV infection, viral hepatitis infections, HIV infection, and syphilis. Specifically, a complete TORCH panel was defined as serological testing covering all seven of the following components: Toxoplasma gondii IgG/IgM, rubella virus IgG/IgM, CMV IgG/IgM, HSV IgG/IgM, hepatitis B surface antigen (HBsAg), HIV antibody testing, and syphilis serology (VDRL or TPHA). Self-reported complete panel status could not be verified against laboratory records, and the distinction between complete and partial testing is based exclusively on participant recall.

Participants could report either the performance of a complete TORCH panel or partial testing consisting of individual investigations recommended during prenatal monitoring.

For descriptive analyses, participants were classified into three categories:

(1) Participants who reported undergoing a complete TORCH panel;
(2) Participants who reported partial TORCH testing;
(3) Participants who reported no TORCH-related investigations.

For logistic regression analyses, the primary outcome variable was recoded into a binary variable:

- Participants who reported undergoing at least one TORCH-related test;
- Participants who reported no TORCH testing.

The binary outcome was coded as 1 (tested) vs. 0 (not tested). The reference category for all regression analyses was the untested group (0). This binary operationalization was chosen to maximize statistical power and interpretability of the logistic regression model, given the relatively small proportion of participants in the “no testing” category (24.4%). It is acknowledged that this approach collapses the distinction between complete and partial TORCH panel testing; sensitivity analyses using a three-category outcome (complete panel vs. partial vs. none) were conducted descriptively and are presented in Table 1.

The study did not evaluate the exact clinical indication for testing, the gestational timing of the investigations, or whether the tests had been recommended by healthcare professionals or directly requested by the patients.

The explanatory variables included maternal age, area of residence, educational level, occupational status, parity, number of prenatal consultations, and participation in prenatal education programs. These variables were selected based on their documented relevance in the literature regarding access to prenatal healthcare services and the utilization of preventive investigations during pregnancy.

### 2.4. Statistical Analysis

Collected data were processed and analyzed using Microsoft Office Excel and IBM^®^ SPSS^®^ Statistics version 23.0. The initial stage included database consistency verification, identification of incomplete or duplicate responses, and logical validation of the analyzed variables.

Categorical variables were described using absolute frequencies and percentages. Chi-square (χ^2^) tests were used for comparisons between groups defined according to the reported type of TORCH testing (complete panel, partial testing, or no testing).

Prior to conducting chi-square analyses, the standard assumptions were verified: observations were independent; the data were organized in mutually exclusive categories; all expected cell frequencies were ≥5 in at least 80% of cells; and no expected cell frequency was <1. For cells in which expected counts fell below 5, Fisher’s exact test was applied as an alternative. These checks were performed for all cross-tabulations reported in Table 1.

Associations between TORCH testing and socio-demographic and obstetrical explanatory variables were evaluated using univariate analyses and multivariable binary logistic regression models. ORs and 95% CIs were calculated to estimate the magnitude of associations.

Multicollinearity was assessed using the Variance Inflation Factor (VIF), with values >10 considered indicative of problematic collinearity. Model fit was evaluated using the Hosmer–Lemeshow goodness-of-fit test (*p* > 0.05 indicating an acceptable fit) and the Nagelkerke R^2^ statistic. In addition, the model’s overall classification accuracy and the area under the receiver operating characteristic (ROC) curve, expressed as the Area Under the Curve (AUC), were reported.

Adjusted odds ratios (aORs) and their corresponding 95% confidence intervals (95% CIs) are presented for each predictor included in the final model. Regression results were expressed as both crude ORs and aORs, together with their respective 95% confidence intervals. The threshold for statistical significance was set at *p* < 0.05 for all inferential analyses.

### 2.5. Ethical Considerations

The study was approved by the Ethics Committee of the Emergency County Hospital “Dr. Constantin Andreoiu”, Ploiești, Romania (No. 41482/9 August 2022), and was conducted in accordance with the ethical principles of the Declaration of Helsinki.

Participation in the study was conditional upon obtaining electronic informed consent prior to questionnaire completion.

## 3. Results

### 3.1. Characteristics of the Study Population

The final analytical sample comprised 1301 pregnant women with complete responses for the primary outcome variable and all explanatory variables. Of these, 983 participants (75.6%) reported having undergone at least one serological investigation for a component of the TORCH panel during pregnancy, while the remaining 318 (24.4%) reported no TORCH-related testing.

The distribution of socio-demographic characteristics showed a predominance of participants residing in urban areas (72.0%) and a high proportion of women with secondary or higher educational levels (93.7%). In addition, most participants were employed at the time of questionnaire completion (75.9%). The largest age group consisted of women aged 20–29 years (51.7%), followed by those aged 30–39 years (43.8%), while participants younger than 20 years represented only 1.2% of the sample.

For a more detailed evaluation of the reported type of prenatal screening, participants were classified into three categories: women who reported undergoing a complete TORCH panel, participants who reported partial TORCH testing, and participants who reported no TORCH-related investigations. Approximately half of the participants (49.3%) reported undergoing a complete TORCH panel, while 26.2% reported partial testing.

The characteristics of participants according to the type of TORCH testing are presented in Table 1.

The results demonstrated statistically significant differences between groups according to educational level, occupational status, and participation in prenatal education programs. Participants with higher educational attainment and those who participated in prenatal education programs more frequently reported undergoing a complete TORCH panel compared with women with lower educational levels or without participation in such programs.

### 3.2. Distribution of Individual Components of the TORCH Panel

In the descriptive analysis of the individual components of the TORCH panel, the most frequently reported investigations were those for *Toxoplasma gondii* and rubella virus, followed by testing for CMV and syphilis. Testing for HSV was reported less frequently compared with the other investigated components.

The distribution of the reported individual TORCH components is presented in Table 2.

Participants could report undergoing one or more serological tests included in the TORCH complex. Since the information was self-reported, and some participants could indicate having undergone a complete TORCH panel while others reported only specific individual investigations, the interpretation of differences between components should be performed with caution.

### 3.3. Analysis of Factors Associated with TORCH Testing Uptake

To evaluate the factors associated with the self-reported performance of testing for one or more infections included in the TORCH complex, univariate analyses and multivariable binary logistic regression models were performed. Variables included in the final model were selected based on clinical relevance and the results of univariate analyses. Variables that were clinically relevant or statistically associated with the outcome in univariate analyses (*p* < 0.20, Wald criterion) were entered simultaneously into the multivariable model using the forced entry method.

The observed associations remained consistent after adjustment for the socio-demographic and obstetrical factors included in the model, suggesting the presence of a socio-educational gradient in the utilization of prenatal screening for infections included in the TORCH complex.

The results of the univariate and multivariable analyses are presented in Table 3.

Primary educational level represented the strongest factor associated with a lower likelihood of self-reported testing for one or more infections included in the TORCH complex (aOR = 0.08; 95% CI: 0.03–0.19; *p* < 0.001). In addition, unemployed occupational status was associated with a lower probability of undergoing these investigations (aOR = 0.70; 95% CI: 0.50–0.99; *p* = 0.045).

Participation in prenatal education programs was associated with a higher likelihood of self-reported testing for one or more infections included in the TORCH panel (aOR = 1.37; 95% CI: 1.04–1.80; *p* = 0.024). In contrast, the number of prenatal consultations did not represent a statistically significant independent predictor in the adjusted model.

Multiparity was independently associated with a lower likelihood of self-reported performance of these investigations (aOR = 0.62; 95% CI: 0.49–0.77; *p* < 0.001), possibly suggesting differences in the utilization of preventive healthcare services among women with previous obstetrical experience.

The interpretation of the findings should be performed in the context of the cross-sectional design and the self-reported nature of the collected data (Table 4).

## 4. Discussion

The results of the present study highlight that undergoing testing for one or more infections included in the TORCH complex during pregnancy was associated predominantly with socio-educational and behavioral factors rather than with the simple frequency of prenatal monitoring. Approximately three-quarters of the participants reported undergoing at least one serological investigation included in the TORCH panel, whereas only about half of the women reported undergoing complete screening. These findings suggest the existence of important differences between access to prenatal care and actual access to standardized preventive interventions for congenital infections [1,2,3].

The analysis of the individual components of the TORCH panel showed that testing for *Toxoplasma gondii* and rubella virus was reported more frequently compared with investigations for CMV or HSV. These differences may reflect variations in clinical practices, medical recommendations, or risk perception associated with certain congenital infections [2]. International literature has frequently reported the lack of standardization of TORCH screening and the variability of prenatal protocols, particularly in the context of differences between national and international recommendations regarding universal versus selective screening strategies [21,22].

One of the most important findings of the study was the strong association between educational level and the self-reported performance of TORCH testing. Participants with a primary educational level showed a significantly lower likelihood of undergoing testing compared with women with higher levels of education. This finding is consistent with the literature demonstrating that maternal educational level significantly influences the utilization of preventive services, health literacy, and informed decision-making capacity during pregnancy [23].

Several studies have shown that maternal education represents one of the most important predictors of prenatal care utilization and participation in prevention programs [21,23]. Women with higher educational attainment tend to use preventive healthcare services more frequently, access validated medical information, and demonstrate better adherence to prenatal recommendations [24,25]. The findings of the present study suggest the existence of a socio-educational gradient in the utilization of prenatal screening for congenital infections, which may contribute to the persistence of inequalities in maternal and neonatal health. The magnitude of the observed association for primary educational level (aOR = 0.08; 95% CI: 0.03–0.19) warrants cautious interpretation. Given that participants with primary education represented a small subgroup within this predominantly highly educated sample, the precision of this estimate may be limited by sparse data effects, and the wide confidence interval should be noted. This association is consistent with the broader literature documenting that lower educational attainment is associated with reduced uptake of preventive healthcare services during pregnancy, although the specific mediating mechanisms—such as differences in health literacy, access to health information, or capacity to navigate healthcare systems—were not measured in the present study and cannot be confirmed from the available data. These proposed pathways remain hypothetical and should be the subject of future targeted investigation. The finding is further consistent with European evidence documenting socio-educational gradients in prenatal screening uptake, although direct comparisons are constrained by differences in study design and healthcare contexts [6,14,23,25].

Occupational status was also independently associated with testing uptake. Unemployed participants showed a lower likelihood of self-reported TORCH investigations compared with employed women. This finding may reflect the impact of socio-economic determinants on access to preventive services and the ability to utilize healthcare during pregnancy [26,27,28]. The literature indicates that socio-economic vulnerability is frequently associated with reduced access to prenatal screening, lower levels of health literacy, and unequal utilization of preventive services [26,27,28]. Several potential mediating mechanisms have been proposed in the literature, including reduced access to occupational health programs, financial barriers, and lower exposure to peer health information networks [26,27,28]. However, as none of these constructs were directly measured in the present study, these pathways remain speculative and should be interpreted as hypotheses for future investigation rather than conclusions supported by the available data.

Another important finding of the study was the negative association between multiparity and testing for one or more infections included in the TORCH complex. Multiparous women showed a lower likelihood of reporting these investigations compared with primiparous women. This result may suggest differences in risk perception and preventive healthcare utilization behaviors among women with previous obstetrical experience [28,29]. Several hypotheses have been proposed in the literature to explain similar patterns, including differences in risk perception between primiparous and multiparous women and competing demands associated with existing childcare responsibilities [29,30,31]. However, these constructs were not directly assessed in the present study, and the mechanisms underlying the observed association cannot be determined from the available data. This finding is consistent with published evidence suggesting that screening behavior may differ between first and subsequent pregnancies, although the present study design does not permit causal conclusions [29,30,31].

Previous studies have shown that multiparous women may perceive pregnancy as less risky in the absence of previous complications and may use prenatal services differently compared with primiparous women [29,30,31]. In this context, previous obstetrical experience may influence the decision to undergo certain investigations considered preventive or optional.

Participation in prenatal education programs was associated with a higher likelihood of self-reported TORCH testing. This finding supports existing evidence showing that educational interventions may improve maternal preventive behaviors and increase the utilization of recommended prenatal services [24,32,33,34]. Prenatal education is considered an important component of modern public health strategies, contributing to increased awareness, reduced pregnancy-related anxiety, and improved adherence to medical recommendations [32,33,34].

In contrast, the number of prenatal consultations did not represent a statistically significant independent predictor of TORCH testing. This finding suggests that the simple frequency of medical contact does not automatically guarantee access to essential preventive interventions. The international literature emphasizes that the evaluation of prenatal care quality should include not only the number of consultations but also their content, access to counseling, and the integration of standardized preventive interventions [35,36].

Congenital infections included in the TORCH complex remain associated with an important risk of fetal and neonatal morbidity, including intrauterine growth restriction, neurological impairment, hearing loss, and other long-term sequelae [1,2,37]. Early identification of maternal infections enables specialized fetal monitoring, parental counseling, and the implementation of appropriate therapeutic measures, contributing to the reduction in perinatal complications [37,38,39,40,41].

The findings of the present study suggest that, in Romania, access to prenatal screening for congenital infections may be significantly influenced by socio-educational and economic factors. In this context, public health strategies should include interventions aimed at reducing social inequalities, developing prenatal education programs, and standardizing recommendations regarding congenital infection screening during pregnancy [25,33,35,41]. From a public health perspective, observational evidence of socio-demographic disparities in preventive healthcare utilization constitutes a recognized basis for policy attention, even in the absence of experimental evidence of causal efficacy [25,26]. The present study identifies three population subgroups—women with lower educational attainment, unemployed women, and multiparous women—that consistently report lower TORCH screening uptake independent of prenatal care frequency. This convergent evidence across predictors, sustained after multivariable adjustment, is consistent with the epidemiological criterion of specificity and aligns with the WHO framework for using observational evidence to prioritize health equity interventions [9]. Translating these findings into policy does not require demonstration of causal mechanisms; it requires only that the identified disparities are plausible, consistent with the broader literature, and addressable through available health system levers [25,35]. Three specific, actionable policy directions are supported by the data. First, the independence of educational level as a predictor—stronger than the number of prenatal consultations—indicates that increasing the quantity of prenatal contacts without addressing content equity is insufficient. Integrating structured TORCH counseling and serological investigation referral into every prenatal consultation, rather than leaving this to individual clinical discretion, would represent a low-cost, high-reach intervention consistent with WHO antenatal care recommendations [9,35]. Second, the protective association of prenatal education program participation (aOR = 1.37; *p* = 0.024) provides direct support for expanding structured prenatal education, particularly targeting low-education and unemployed populations through community outreach and primary care integration [32,33]. Third, the independent association of multiparity with reduced screening uptake supports the introduction of systematic re-screening prompts for multiparous women in clinical workflows, acknowledging that prior obstetric experience does not guarantee equivalent preventive investigation coverage in subsequent pregnancies [29,30,31]. These policy directions are proportionate to the level of evidence available, do not require causal proof, and are already endorsed by comparable European public health frameworks [6,9]. The Romanian context—characterized by the absence of a uniform national TORCH screening protocol and documented regional disparities in prenatal care access [14,17]—makes equity-focused standardization particularly relevant and feasible within existing maternal health infrastructure.

The interpretation of the findings should be performed in the context of the methodological limitations of the study. The cross-sectional design does not allow the establishment of causal relationships between the investigated variables and TORCH testing uptake. In addition, the data were self-reported and collected exclusively online, aspects that may introduce selection bias toward more digitally connected, urban, and highly educated participants, which likely results in an overestimate of population-level screening coverage. Furthermore, the classification of testing status is based exclusively on participant recall without objective verification against medical records or laboratory registries, which may introduce non-differential misclassification. These limitations constrain the external validity of the quantitative estimates but do not negate the identification of disparities in screening uptake across socio-demographic subgroups, which are internally consistent and aligned with the broader European evidence base [6,14,23]. The study also presents several methodological strengths that support the robustness of the reported associations. The sample size (*n* = 1301) substantially exceeds the a priori estimated minimum required for adequate statistical power. The multivariable logistic regression model controlled simultaneously for maternal age, area of residence, educational level, occupational status, parity, number of prenatal consultations, and prenatal education participation, enabling the estimation of independent effects for each predictor. The model demonstrated satisfactory goodness-of-fit (Hosmer-Lemeshow test *p* > 0.05) and adequate overall discriminatory ability. The consistency of the socio-educational gradient across multiple predictors, the magnitude of the association with primary educational level, and the protective effect of prenatal education participation collectively constitute a coherent pattern that is transferable to health system planning, even in the absence of causal proof [25,35].

## 5. Conclusions

This cross-sectional study reports that prenatal serological screening for infections included in the TORCH complex—as assessed using an expanded Romanian panel including hepatitis B and HIV in addition to the classical TORCH agents, and based exclusively on participant self-report without laboratory verification—was reported by 75.6% of pregnant women recruited online in Romania, yet complete TORCH panel coverage was achieved by only approximately half (49.3%) of participants. Given the online recruitment strategy and the predominantly urban and highly educated sample, these uptake rates likely overestimate population-level coverage and should be interpreted with caution. Primary educational level was the strongest independent predictor of reduced testing uptake (aOR = 0.08; 95% CI: 0.03–0.19; *p* < 0.001); given the small number of participants with primary education in this sample, this estimate should be interpreted with caution as it may be subject to sparse data effects. Unemployment (aOR = 0.70; *p* = 0.045) and multiparity (aOR = 0.62; *p* < 0.001) were additional independent factors associated with lower screening uptake. Conversely, participation in prenatal education programs was independently associated with higher testing rates (aOR = 1.37; *p* = 0.024).

Notably, the number of prenatal consultations was not an independent predictor of TORCH testing (aOR = 1.02; *p* = 0.771), indicating that the frequency of clinical contact alone does not guarantee the delivery of standardized preventive investigations. These findings collectively point to a quality gap in prenatal care that disproportionately affects women with lower educational and socio-economic status.

These findings have direct relevance for health system planning in Romania, within the recognized framework for using observational evidence to guide equity-focused public health interventions. Acknowledging that the cross-sectional, self-reported design does not establish causal efficacy of specific interventions, the consistency and magnitude of the identified socio-demographic disparities support three priority directions. At the health system level, the absence of a uniform national protocol for TORCH-related prenatal investigations creates the structural conditions under which individual socio-demographic disadvantage translates into differential access to preventive care; standardizing investigation referral pathways within existing prenatal care frameworks—without requiring mandatory programme-level implementation—would be a proportionate and feasible first step. At the clinical level, the null finding for the number of prenatal consultations as a predictor of screening uptake, combined with the significant protective effect of prenatal education participation, indicates that reforming the content of prenatal consultations to include systematic infectious disease counseling and investigation referral is a higher-yield strategy than increasing consultation frequency alone. At the community level, multiparous women and unemployed women represent identifiable, reachable groups with consistently lower screening uptake that could be targeted through proactive outreach integrated into primary care and community midwifery services. Future research should employ registry-based and interventional designs to evaluate the impact of these targeted strategies on screening coverage, congenital infection detection rates, and perinatal outcomes at the population level.

## Figures and Tables

**Table 1:** Characteristics of Participants According to the Type of Testing for Infections Included in the TORCH Complex.

Characteristic	Complete TORCH Panel *n* (%)	Partial Testing *n* (%)	No Testing *n* (%)	*p*-Value
Total participants	642 (49.3)	341 (26.2)	318 (24.4)	-
Maternal age < 20 years	10 (62.5)	4 (25.0)	2 (12.5)	**0.031**
Urban residence	482 (51.5)	229 (24.5)	225 (24.0)	0.684
Higher education	501 (51.3)	252 (25.9)	220 (22.6)	**<0.001**
Employed occupational status	512 (51.9)	251 (25.4)	224 (22.7)	**0.041**
Participation in prenatal education	388 (54.1)	179 (24.9)	151 (21.0)	**0.028**

Note: Values are expressed as *n* (%). (-) = not applicable; no chi-square test was performed for the total row, as it represents marginal counts. Statistical significance was evaluated using the chi-square (χ^2^) test. Statistically significant differences (*p* < 0.05) were observed for: maternal age < 20 years (*p* = 0.031); higher educational level (*p* < 0.001); employed occupational status (*p* = 0.041); participation in prenatal education programs (*p* = 0.028). Urban residence did not reach statistical significance (*p* = 0.684). Bold *p*-values indicate statistically significant associations.

**Table 2:** Reported Distribution of Individual Components of the TORCH Panel.

Reported TORCH Component	*N* % of Tested Participants
Toxoplasma gondii	912 (92.8)
Rubella virus	884 (89.9)
Cytomegalovirus (CMV)	741 (75.4)
Syphilis	693 (70.5)
Herpes simplex virus (HSV)	418 (42.5)

Note: Percentages are calculated among participants who reported undergoing at least one TORCH-related investigation (*n* = 983). Values represent the proportion of tested participants who reported each specific serological test. Since participants could report more than one component, percentages do not sum to 100%. No formal statistical comparison between individual TORCH components was performed in this table; data are presented descriptively.

**Table 3:** Factors Associated with the Self-Reported Performance of Testing for One or More Infections Included in the TORCH Complex.

Predictor	Reference Category	Crude OR (95% CI)	*p*-Value	Adjusted OR (95% CI)	*p*-Value
Maternal age < 20 years	20–29 years	1.74 (1.01–3.02)	0.041	1.92 (1.10–3.34)	0.023
Maternal age 30–39 years	20–29 years	0.79 (0.61–0.98)	0.029	0.71 (0.54–0.93)	0.013
Maternal age ≥ 40 years	20–29 years	1.11 (0.82–1.59)	0.118	1.25 (0.89–1.77)	0.094
Rural residence	Urban	0.96 (0.74–1.23)	0.711	0.94 (0.71–1.25)	0.700
Primary educational level	Higher education	0.10 (0.04–0.22)	<0.001	0.08 (0.03–0.19)	<0.001
Secondary educational level	Higher education	0.94 (0.73–1.21)	0.637	0.97 (0.75–1.26)	0.960
Unemployed occupational status	Employed	0.76 (0.58–0.98)	0.039	0.70 (0.50–0.99)	0.045
Multiparity	Primiparity	0.66 (0.53–0.81)	<0.001	0.62 (0.49–0.77)	<0.001
Number of prenatal consultations	Continuous variable	1.01 (0.91–1.14)	0.602	1.02 (0.90–1.16)	0.771
Participation in prenatal education	No	1.29 (1.01–1.67)	0.034	1.37 (1.04–1.80)	0.024

**Table 4:** Forest plot of aOR and 95% CI for predictors of self-reported TORCH screening uptake during pregnancy (multivariable binary logistic regression; *n* = 1301).

Predictor Variable	Forest Plot (log Scale: 0.05…10.0)	aOR	95% CI	*p*-Value
Primary education (vs. higher education)	–––––■–––––– |	0.08	0.03–0.19	**<0.001**
Multiparity (vs. primiparity)	–■––|	0.62	0.49–0.77	**<0.001**
Unemployed (vs. employed)	––■–|	0.70	0.50–0.99	**0.045**
Age 30–39 years (vs. 20–29 years)	–■–|	0.71	0.54–0.93	**0.013**
Prenatal education (vs. none)	|–■––	1.37	1.04–1.80	**0.024**
Age < 20 years (vs. 20–29 years)	|–––■–––	1.92	1.10–3.34	**0.023**
Rural residence (vs. urban)	––■–	0.94	0.71–1.25	0.700
Age ≥ 40 years (vs. 20–29 years)	–|■––	1.25	0.89–1.77	0.094
Secondary education (vs. higher education)	––■–	0.97	0.75–1.26	0.960
Prenatal consultations (continuous)	–■–	1.02	0.90–1.16	0.771

Note: aOR = adjusted odds ratio; CI = confidence interval. The forest plot column uses a logarithmic scale (0.05–10.0). The vertical bar (“|”) marks OR = 1.0; ■ = point estimate; – = 95% CI. Bold values indicate statistical significance (*p* < 0.05). Multivariable binary logistic regression; *n* = 1301.

## Data Availability

The original contributions presented in this study are included in the article. Further inquiries can be directed to the corresponding author.

## References

[1] Neu N., Duchon J., Zachariah P. (2015). TORCH infections. Clin. Perinatol..

[2] Maldonado Y.A., Nizet V., Klein J.O., Remington J.S., Wilson C.B., Wilson C.B., Nizet V., Maldonado Y.A. (2016). Current concepts of infections of the fetus and newborn infant. Remington and Klein’s Infectious Diseases of the Fetus and Newborn Infant.

[3] World Health Organization (2021). Congenital Infections: Prevention, Diagnosis and Management.

[4] American College of Obstetricians and Gynecologists’ Committee on Practice Bulletins—Obstetrics, Committee on Genetics, Society for Maternal-Fetal Medicine (2020). Screening for Fetal Chromosomal Abnormalities: ACOG Practice Bulletin, Number 226. Obstet. Gynecol..

[5] Rawlinson W.D., Boppana S.B., Fowler K.B., Kimberlin D.W., Lazzarotto T., Alain S., Daly K., Doutré S., Gibson L., Giles M.L. (2017). Congenital cytomegalovirus infection in pregnancy and the neonate: Consensus recommendations for prevention, diagnosis, and therapy. Lancet Infect. Dis..

[6] European Centre for Disease Prevention and Control (2022). Congenital Infections in Europe: Epidemiological Update.

[7] Schneider M.O., Faschingbauer F., Kagan K.O., Groß U., Enders M., Kehl S., AGG Section Maternal Diseases (2023). Toxoplasma gondii Infection in Pregnancy—Recommendations of the Working Group on Obstetrics and Prenatal Medicine (AGG—Section on Maternal Disorders). Geburtshilfe Frauenheilkd..

[8] Rac M.W.F., Stafford I.A., Eppes C.S. (2020). Congenital syphilis: A contemporary update on an ancient disease. Prenat. Diagn..

[9] World Health Organization (2016). WHO Recommendations on Antenatal Care for a Positive Pregnancy Experience.

[10] Mazzitelli M., Micieli M., Votino C., Visconti F., Quaresima P., Strazzulla A., Torti C., Zullo F. (2017). Knowledge of Human Cytomegalovirus Infection and Prevention in Pregnant Women: A Baseline, Operational Survey. Infect. Dis. Obstet. Gynecol..

[11] Centers for Disease Control and Prevention (2023). Screening Recommendations for Congenital Infections During Pregnancy.

[12] Peeling R.W., Mabey D., Kamb M.L., Chen X.S., Radolf J.D., Benzaken A.S. (2017). Syphilis. Nat. Rev. Dis. Prim..

[13] Nutbeam D. (2000). Health literacy as a public health goal: A challenge for contemporary health education and communication strategies. Health Promot Int..

[14] Radoi C.L., Zlatian O., Balasoiu M., Giubelan L., Stoian A.C., Dragonu L., Neacsu A., Iliescu D.G. (2023). Seroprevalence of Infections with TORCH Agents in Romania: A Systematic Review. Microorganisms.

[15] Cernat E.M., Dima A., Popescu C., Neagu A., Betianu C., Moga M., Manolescu L.S.C., Barbilian A. (2024). Anterior Intercondylar Notch Geometry in Relation to the Native Anterior Cruciate Ligament Size. J. Clin. Med..

[16] Manolescu L., Marinescu P. (2013). Sex differences in HIV-1 viral load and absolute CD4 cell count in long term survivors HIV-1 infected patients from Giurgiu, Romania. Romanian Rev. Lab. Med..

[17] Ministerul Sănătăţii [Ministry of Health], Romania (2008). Order No. 1982/2008 on the Approval of the Monitoring Programme for the Evolution of Pregnancy and Puerperium.

[18] Fowler K., Mucha J., Neumann M., Lewandowski W., Kaczanowska M., Grys M., Schmidt E., Natenshon A., Talarico C., Buck P.O. (2022). A systematic literature review of the global seroprevalence of cytomegalovirus: Possible implications for treatment, screening, and vaccine development. BMC Public Health.

[19] Binda S., Pellegrinelli L., Terraneo M., Caserini A., Primache V., Bubba L., Barbi M. (2016). What people know about congenital CMV: An analysis of a large heterogeneous population through a web-based survey. BMC Infect. Dis..

[20] Vauloup-Fellous C., Picone O., Cordier A.G., Parent-du-Châtelet I., Senat M.V., Frydman R., Grangeot-Keros L. (2009). Does hygiene counseling have an impact on the rate of CMV primary infection during pregnancy? Results of a 3-year prospective study in a French hospital. J. Clin. Virol..

[21] American College of Obstetricians and Gynecologists (2021). Routine tests during pregnancy. ACOG Practice Bulletin.

[22] Adler N.E., Newman K. (2002). Socioeconomic disparities in health: Pathways and policies. Health Aff..

[23] Lindquist A., Kurinczuk J.J., Redshaw M., Knight M. (2015). Experiences, utilisation and outcomes of maternity care in England among women from different socio-economic groups. BMC Pregnancy Childbirth.

[24] Gage A.J. (2007). Barriers to the utilization of maternal health care in rural Mali. Soc. Sci. Med..

[25] Braveman P., Gottlieb L. (2014). The social determinants of health: It’s time to consider the causes of the causes. Public Health Rep..

[26] Marmot M. (2005). Social determinants of health inequalities. Lancet.

[27] Heaman M.I., Sword W., Elliott L., Moffatt M., Helewa M.E., Morris H., Gregory P., Tjaden L., Cook C. (2015). Barriers and facilitators related to use of prenatal care by inner-city women. J. Obstet. Gynecol. Neonatal Nurs..

[28] Beeckman K., Louckx F., Putman K. (2010). Determinants of the number of antenatal visits in a metropolitan region. BMC Public Health.

[29] Partridge S., Balayla J., Holcroft C.A., Abenhaim H.A. (2012). Inadequate prenatal care utilization and risks of infant mortality and poor birth outcome. BMC Pregnancy Childbirth.

[30] Holcomb D.S., Pengetnze Y., Steele A., Karam A., Spong C., Nelson D.B. (2021). Geographic barriers to prenatal care access and their consequences. Am. J. Obstet. Gynecol. MFM.

[31] Gagnon A.J., Sandall J. (2007). Individual or group antenatal education for childbirth or parenthood, or both. Cochrane Database Syst. Rev..

[32] World Health Organization (2015). Recommendations on Health Promotion Interventions for Maternal and Newborn Health.

[33] Rosenstock I.M. (1974). Historical origins of the health belief model. Health Educ. Monogr..

[34] Carroli G., Rooney C., Villar J. (2001). How effective is antenatal care in preventing maternal mortality and serious morbidity?. Paediatr. Perinat. Epidemiol..

[35] Tunçalp Ӧ., Pena-Rosas J.P., Lawrie T., Bucagu M., Oladapo O.T., Portela A., Gülmezoglu A.M. (2017). WHO recommendations on antenatal care for a positive pregnancy experience—Going beyond survival. BJOG.

[36] Boppana S.B., Pass R.F., Britt W.J., Stagno S., Alford C.A. (1992). Symptomatic congenital cytomegalovirus infection: Neonatal morbidity and mortality. Pediatr. Infect. Dis. J..

[37] Pomares C., Montoya J.G. (2016). Laboratory Diagnosis of Congenital Toxoplasmosis. J. Clin. Microbiol..

[38] United Nations Children’s Fund (UNICEF) (2021). Antenatal Care and Maternal Health Services.

[39] Marinescu P., Manolescu L.S.C. (2012). Association of hepatitis B infection in patients with HIV Encephalopathy. Rom. Biotechnol. Lett..

[40] Preda M., Chivu R.D., Ditu L.M., Popescu O., Manolescu L.S.C. (2024). Pathogenesis, Prophylaxis, and Treatment of Candida auris. Biomedicines.

[41] Dragomirescu C.C., Lixandru B.E., Coldea I.L., Palade A.M., Baltoiu M., Dinu S., Cristea V.C., Manolescu L., Popa M.I. (2017). Comparative analysis of different phenotypic and molecular methods used for the taxonomic identification of *Corynebacterium* spp. isolated from clinical samples in Romania. Rom. Biotechnol. Lett..

